# A rapid review of recent advances in diagnosis, treatment and vaccination for COVID-19

**DOI:** 10.3934/publichealth.2021011

**Published:** 2021-02-01

**Authors:** Srikanth Umakanthan, Vijay Kumar Chattu, Anu V Ranade, Debasmita Das, Abhishekh Basavarajegowda, Maryann Bukelo

**Affiliations:** 1 Department of Paraclinical Sciences, Faculty of Medical Sciences, The University of the West Indies, St. Augustine, Trinidad and Tobago, West Indies; 2 Department of Medicine, Temerty Faculty of Medicine, University of Toronto, Toronto, ON M5G2C4, Canada; 3 Division of Occupational Medicine, St. Michael's Hospital, Unity Health Toronto, Toronto, ON M5C 2C5, Canada; 4 Department of Basic Medical Sciences, College of Medicine, University of Sharjah, PO Box 27272, USA; 5 Department of Pathology and Laboratory Medicine, Nuvance Health Danbury Hospital Campus, Connecticut, Zip 06810, USA; 6 Department of Transfusion Medicine, Jawaharlal Institute of Postgraduate Medical Education and Research, Puducherry, PIN-605006, India; 7 Department of Anatomical Pathology, Eric Williams Medical Sciences Complex, North Central Regional Health Authority, Trinidad and Tobago, West Indies

**Keywords:** coronavirus, genome, diagnosis, therapy, vaccine, Antigen test, BCG, Reverse Transcription Polymerase Chain Reaction

## Abstract

COVID-19 is caused by SARS-CoV-2, which originated in Wuhan, Hubei province, Central China, in December 2019 and since then has spread rapidly, resulting in a severe pandemic. The infected patient presents with varying non-specific symptoms requiring an accurate and rapid diagnostic tool to detect SARS-CoV-2. This is followed by effective patient isolation and early treatment initiation ranging from supportive therapy to specific drugs such as corticosteroids, antiviral agents, antibiotics, and the recently introduced convalescent plasma. The development of an efficient vaccine has been an on-going challenge by various nations and research companies. A literature search was conducted in early December 2020 in all the major databases such as Medline/PubMed, Web of Science, Scopus and Google Scholar search engines. The findings are discussed in three main thematic areas namely diagnostic approaches, therapeutic options, and potential vaccines in various phases of development. Therefore, an effective and economical vaccine remains the only retort to combat COVID-19 successfully to save millions of lives during this pandemic. However, there is a great scope for further research in discovering cost-effective and safer therapeutics, vaccines and strategies to ensure equitable access to COVID-19 prevention and treatment services.

## 1. Introduction

Coronoviridae consists of a group of RNA viruses named Coronaviruses (CoV). CoV is a non-segmented RNA virus that is enveloped [1]. Historically, they have caused varying forms of infections affecting humans and other mammals. HCoV-229E, HCoV-OC43, HCoV-NL63, HCoV-HKU1 are milder forms of the virus than SARS-CoV MERS-CoV, which have resulted in higher case fatality rates [2].

The genomic component of SARS-CoV-2 encodes four major structural proteins: spike (S), membrane (M), envelope (E), and nucleocapsid (N). Besides, it encodes numerous other nonstructural and accessory proteins. This virus's pathogenicity is mainly dependent on virus-host interaction between the virus S protein and host membrane receptor angiotensin-converting enzyme 2 (ACE2) [3]. The SARS-CoV-2 binding affinity with ACE2 is higher than other CoV species, resulting in higher transmission, replication, and virulence rate [4]. These features have caused SARS-CoV-2 to emerge as a global pandemic, making vaccine research an integral part of disease containment [5]. This article is aimed to give a detailed insight into the recent trends of diagnosis and therapeutic options for COVID-19. We further discuss the on-going vaccine trials and the challenges encountered in the investigational vaccine for COVID-19. Due to the rapidly evolving nature of COVID-19, the readers are requested to update themselves with the nature of change with this particular type of Coronavirus.

## 2. Methodology

**Figure 1.**
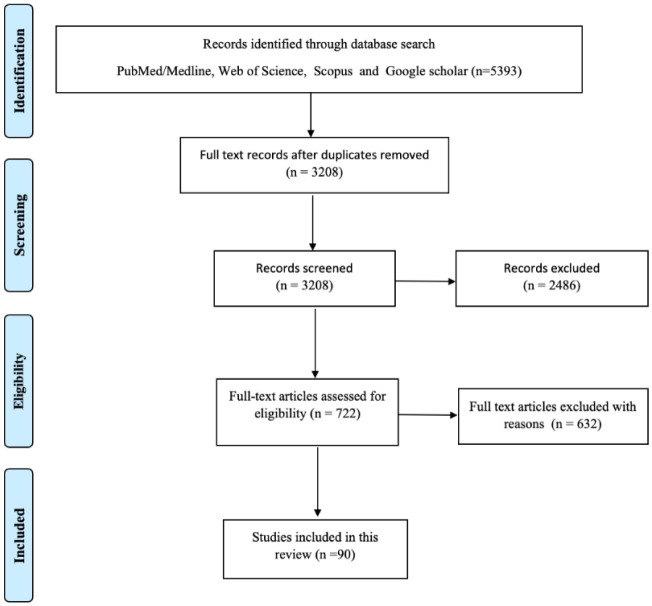
Flow chart for the literature search.

A literature search was done in early December 2020. All the main databases such as Medline/PubMed, Web of Science, Scopus, and Google Scholar for the keywords “Diagnostics” or “Therapeutics” or “Vaccines” or “Case management” and “COVID-19”. All the relevant articles published in 2020 since the epidemic (from January 1–December 4, 2020) with available full text were collected, and the duplicates were removed. After assessing the articles based on the eligibility criteria, a total of 90 research articles (Metaanalysis-5; Clinical trials-32 and Systematic reviews-53) were included. No hand search was performed. We have sorted the publications into the three main sections: addressing 1) Diagnostics for COVID-19, 2) Therapeutics, and 3) Potential Vaccines for COVID-19. The significant findings and options found to be useful in the case management from various countries and clinical setups are described in the results section. The literature search is depicted in the flow chart shown in Figure 1.

## 3. Results

### 3.1. Diagnostic approaches for COVID-19

**Figure 2.**
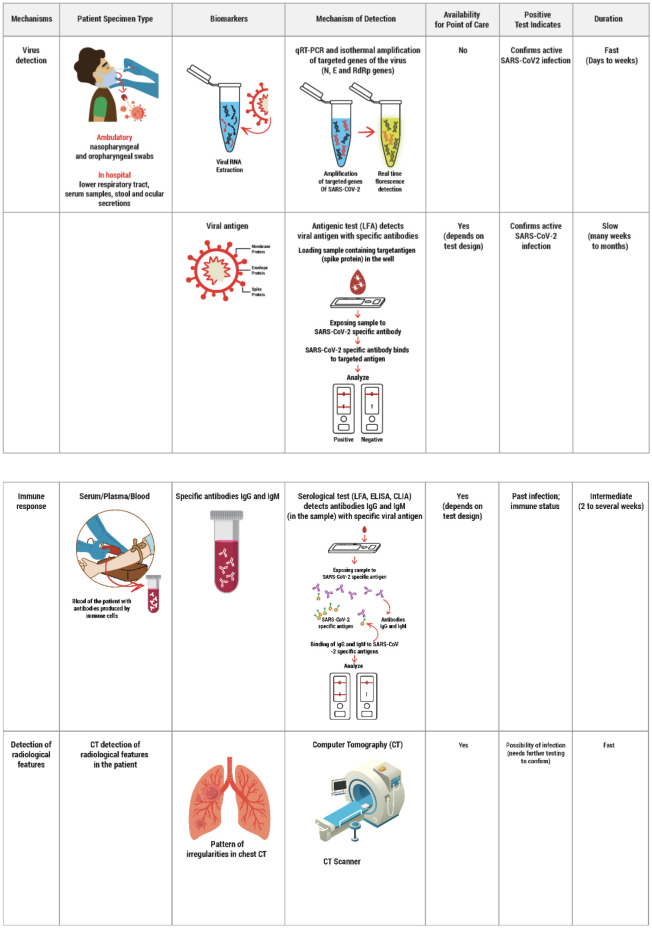
Various patient sample collection methods, mechanism of SARS-CoV2 detection, and interpretation of the test results.

Patients infected with SARS-CoV-2 have predominantly presented with fever, cough, and malaise. Other symptoms targeting the respiratory, gastrointestinal, and urinary systems have been documented based on the patient's age and persisting comorbidities [6]. The non-specific clinical manifestation of SARS-CoV-2 provides a need for rapid diagnosis to have an effective control of this raging disease. This can be attained by sensitive and specific diagnostic tools such as gene sequencing, electron microscopy, and cell culture methods [7]. Real-time reverse transcriptase-polymerase chain reaction (RT-PCR) has been the most common rapid diagnostic test in diagnosing SARS-CoV-2, as it couples the principle of transcription and amplification, thereby providing a high specificity rate [8]. Specimens for virus detection vary from nasopharyngeal and oropharyngeal swabs and broncho-alveolar lavage, sputum to stool, urine, and blood, depending on the clinical manifestation of the patients [9]. Certain vital precautions to be taken while specimen collection are: 1) Personal protective equipment (PPE) is essential as the swab collection poses a high transmission risk due to close contact between the health worker and the suspected patient, 2) Storage and transportation should be done effectively to prevent false-negative and false-positive results, 3) Swab collection should be done cautiously in patients having low platelet count as it may result in easy bruising and bleeding at the collection sites [10],[11]. The following figured table (Figure 2) depicts various patient sample collection methods, the mechanism of SARS-CoV2 detection, and interpretation of the test results. The diagnostic tests to detect the on-going or past viral infection can be categorized into four types.

#### 3.1.1. Nucleic acid test (NAT)

It is currently the gold standard test to detect SARS-CoV-2. It uses Reverse Transcription Polymerase Chain Reaction (RT-PCR) to detect the viral RNA from samples obtained through the specimen sites [12]. RT-PCR is a sensitive and specific technique that involves sequential binding on the genetic material that targets a specific pathogen of interest and amplifies that specified region with every cycle in an ideal reaction condition [13]. A fluorescent emission occurs in each cycle, and the test is considered positive when its intensity reaches a threshold. The false-positive cases are disparaged due to the amplification and analysis occurring concurrently. The SARS-CoV-2 genes targeted to date include N, E, and RNA dependent RNA polymerase (RdRp). The occurrence of these genes in the test will be considered positive [14]. The test's processing time may take approximately 45 minutes and is currently the fastest test [15]. Abbott's ID NOW™, with their latest development in technique, targets the RdRp gene and confirms the test in less than 13 minutes. This has received FDA emergency use authorization to be used in epicentres of the outbreak. However, the major limitation is that the test can analyze only one sample at a time [16].

#### 3.1.2. Antigen test

This test detects the presence of SARS-CoV-2 nucleocapsid protein antigen on the viral surface from nasopharyngeal and oropharyngeal swabs [17]. This test relies on specified monoclonal antibodies (MAb) to bind to specific viral antigens in fluid samples. It can be detected using calorimetric enzyme immunoassay, enhanced chemiluminescent immunoassay, and the most recent, affordable, rapid, and user-friendly fluorescence lateral flow assay (LFA). The structural proteins such as spike (S), membrane (M), envelope (E), and nucleocapsid (N) proteins of the coronaviruses are the key foci in the SARS-CoV-2 virus for ensuing antigen-based tests. However, due to its low detection sensitivity and specificity than PCR, the antigen-based tests are not considered a gold standard in the diagnosis of COVID-19 [18],[19].

#### 3.1.3. Antibody test

It detects specific antibodies (IgG and IgM) produced against SARS-CoV-2 in the serum, plasma, or whole blood of the host in response to the viral infection. It plays a vital role in detecting the infection during the later phases and identifying people with immunity against the disease. It can also be useful to monitor the disease's progression and in the development of a vaccine [20]. Enzyme-linked immunosorbent assay (ELISA), chemiluminescence assay (CLIA), and LFA are a few of the most commonly used assay for the antibody test. Lateral flow assay is an emerging, inexpensive, and accessible antibody detecting test for the diagnosis of SARS-CoV-2. It is highly specific but less sensitive compared to RT-PCR. Severe cases presenting with high viremic load are easily detected by this method providing an easy flag for patients with poor prognosis [21].

#### 3.1.4. Image-based test

RT-PCR is currently considered the gold standard to detect the SARS CoV-2. However, its low sensitivity for early detection of the disease due to low viral load, coupled with limited availability of the test kits in various parts of the world. These factors have made C.T. chest being accepted as an additional tool in diagnosing patients suspected with COVID-19 [22]. Since its introduction into the guidelines for the diagnosis of treatment of COVID-19, CT scan has played a pivotal role in the diagnosis and treatment of the disease. Ground glass opacities are the predominant pattern of abnormalities seen after the onset of symptoms followed by the mixed pattern. In most patients, the location of the lesions is bilateral and sub-segmental. However, these abnormal findings may be non-specific as they may overlap with viral pneumonia of different etiologies [23].

Each of these above tests plays a pivotal and complementary role in various COVID-19 pandemic progression stages.

### 3.2. Therapeutic options for COVID-19

Symptomatic cases should be admitted to hospitals and treated effectively. Treatment should always be initiated in designated hospitals that follow strict isolation and prevention protocol measures. General supportive therapy, including oxygenation and fluid management, is directed towards maintaining the patient's hemodynamic status and vital signs.

Numerous management modes have been proposed globally to combat COVID-19, of which few treatment options have been effective and also approved. Herein, these drugs' roles are reviewed to identify their potency in the treatment of symptomatic cases.

#### 3.2.1. Corticosteroids

Corticosteroids have been restricted in managing cases infected with CoV due to their immunosuppression effects. Based on the prospective recovery trial conducted in UK-NHS hospitals, dexamethasone 6mg/day (oral/IV) has shown a significant decline in mortality (35%) in patients who were on invasive mechanical ventilation. It has also demonstrated an early recovery and shorter hospitalization in COVID-19 patients [24]. These effects are primarily through its anti-inflammatory properties by reducing the collection of exudates within the lung alveolar spaces, thereby preventing diffuse alveolar damage and consequently reducing the risk of developing acute respiratory distress syndrome (ARDS) [25]. Adverse effects include dysglycemia in diabetics, hypo-pituitary-adrenal axis suppression in patients with pre-existing adrenal hypofunction, and lipotoxicity. These adverse effects should be observed and corrected on time [24].

#### 3.2.2. Antiviral therapy

Remdesivir is a broad-spectrum antiviral drug used effectively in treating patients infected with the Ebola virus, MERS-CoV, and SARS-CoV-1. It efficiently delivers a monophosphate nucleic acid analog into the cell and further undergoes an active transformation, and selectively inhibits RdRp [26]. Serious adverse effects include acute renal failure, sepsis, and multi-organ failure. For these reasons, the use of Remdesivir is permitted only to treat adults and children with laboratory-confirmed COVID-19; and in those patients requiring mechanical ventilation or extracorporeal membrane oxygenation (ECMO) [27].

Chloroquine/ hydroxychloroquine (CQ/HCQ) is an antimalarial drug that also provides post-transcriptional inhibition in HIV patients. The anti-HIV effects are glycoprotein (G.P.) 120 encoding, low _P_.H., and coating/uncoating of virus particles [28]. CQ/HCQ produces numerous toxic effects, and their wide use has shown to cause sudden cardiac death in cases when used as HCQ-Azithromycin combination. Hence CQ/HCQ should be used with caution until a well-established report showing its efficacy against CoV is published [29].

Lopinavir/ritonavir are protease inhibitors used as anti-HIV drugs. These drugs are cheap, readily available, and are listed as essential drugs by WHO. In COVID-19 patients, these drugs have shown no significant differences in mortality rates. However, they have been shown to have reduced the hospital stay and reduced renal and pulmonary complications in COVID-19 patients [30]. They inhibit SARS-CoV-3CL pro (chymotrypsin-like protease enzyme), responsible for viral replication [31].

Ivermectin has a multifaceted property with its anti-microbial and anti-cancer actions. Its antiviral effects are mainly on RNA group of viruses such as Zika, West Nile, Yellow fever, type-1 HIV, and SARS-CoV-2 [32]. Based on a recent in-vitro study, Ivermectin inhibits importin receptors responsible for viral protein transmission into the host cell nucleus within 48 hours of initiating ivermectin treatment. Due to limited clinical trials undertaken to date, ivermectin is still pending approval for its safety and efficacy in treating COVID-19 patients [33].

Favipiravir is a purine nucleoside analogue developed in Japan and is known for its antiviral properties against influenza A and B. It acts by selective dysregulation of viral RNA replication, inducing mutagenic destruction of RNA viruses. This drug has exerted sufficient efficacy in vitro Vero E6 cells infected with SARS-CoV-2 [34]. This effect occurs at high concentrations. Clinical trials conducted in China and Japan have shown a higher recovery rate with a significantly shorter duration of fever and cough symptoms [35].

Oseltamivir (Tamiflu) is a neuraminidase inhibitor approved for treating influenza A and B. This drug is still under clinical trial for COVID-19 treatment approval and is used in combination with Chloroquine and Favipiravir [36].

#### 3.2.3. Antibiotics

The role of antibiotics in COVID-19 patients is mainly restricted to patients with co-infection or those suffering from secondary bacterial pneumonia. It should be used only in patients presenting with severe clinical respiratory insufficiency. The antibiotics should be given for a maximum of 5 days, following which the patient should be re-evaluated with bacteriological tests and C.T. scans [37],[38].

#### 3.2.4. Blood derived products

Recent interest generated in the use of convalescent plasma as a possible therapeutic option in critically ill COVID-19 is supported by the U.S. and has received FDA approval to use the plasma from patients who have recovered from COVID-19.

The proposed mechanisms of action of convalescent plasma in COVID-19 are immune-mediated suppression of viremia by neutralization, inflammatory response modification by cytokines, antibody-dependent cellular cytotoxicity, complement activation, and phagocytosis (ADCP). The non-immune mechanism is involved in the restoration of coagulation factors [39],[40].

Collection: Convalescent plasma is generally collected by apheresis technology from patients who have had RT-PCR proven infection of COVID-19 and recovered symptomatically for at least 14 days and tested negative with fair certainty (demonstration of two non-reactive NAT for SARS-CoV-2 performed at an interval of 24 hours apart on nasopharyngeal swabs). The donor should be otherwise eligible for blood/plasma donation under local requirements and standards and tested negative for Transfusion transmitted infections by approved serological/molecular tests. Up to 600 ml can be collected and repeated seven days apart in compliance with applicable regulatory limits. To avoid transfusion-related acute lung injury (TRALI), multiparous women are generally excluded from donation [41].

Preparation: The product is characterized by determining titers of total and neutralizing anti-SARS-CoV-2 antibodies before use and is stored frozen at preferably beyond −40°C. Pathogen inactivation of plasma wherever feasible is desirable using a licensed technology to control residual risks of infectious disease, including allaying fears of SARS-CoV-2 superinfections [41].

Transfusion: The frozen plasma is thawed and transfused to the patient after ensuring ABO compatibility. Generally, plasma from two different donors is advisable to deliver diverse antibodies. Usually, a dose of 200 mL is recommended, followed by one or two additional doses of 200 mL based on the severity of the recipients' disease and tolerability.

A systematic review of the Cochrane database available as of date showed that there was only one randomized controlled trial (RCT), which was stopped early, and three controlled non-randomized studies of interventions. There are almost 98 on-going studies evaluating convalescent plasma, of which 50 are randomized. These studies' have uncertain effects on mortality rates, and improvement of clinical symptoms are assessed by the need for respiratory support for 7 to 28 days. The majority of adverse reactions reported were allergic or respiratory-related complications [42].

There is no much-documented evidence to suggest that this therapy is beneficial to prevent patients presenting with mild symptoms or those with severe illness [43]. About ensuring a high likelihood of achieving sufficiently high S-RBD (spike protein, receptor binding domain)-specific IgG titers, this procedure is initiated in donors after 28 days from onset of fever. No significant correlation to age, gender, and ABO blood type of donors is noted though it is speculated that males with higher age had better antibody titers [44].

Mesenchymal stem cell therapy (MSCT) extends a promising approach towards alleviating the deleterious effects of the infection in COVID-19 patients. This therapy has been shown to decrease the expression of pro-inflammatory cytokines and in repairing the damaged tissues. They can protect alveolar endothelium by lung aggregation, improvement in the microenvironment, rearrangement of immune cell subsets' functions, regulation of inflammatory cytokines, and T and B lymphocytes' inhibition [45],[46]. Currently, there are no approved MSCT for preventing or treating COVID-19 patients, but there are on-going clinical trials [47]. MSCT has a high proliferation rate, less invasive, and easily obtained from the fat, placenta, and stored easily.

#### 3.2.5. Immune-based therapy

Tocilizumab is a recombinant humanized monoclonal antibody directed against the IL-6 receptor. It is currently used to treat rheumatoid arthritis, giant cell arteritis, and in cases of life-threatening cytokine storm conditions. A retrospective study showed tocilizumab reduced the mortality rates, ICU admissions and also lowered the risk of invasive mechanical ventilation in severe COVID-19 patients [48],[49].

Baricitinib is a selective JAK1/2 kinase inhibitor used in the treatment of rheumatoid arthritis and psoriatic arthritis. Its additional cyclin-G-kinase binding action prevents the occurrence of immune-mediated ARDS in severe COVID-19 cases [50],[51].

Ruxolitinib and anakinra are the other immunomodulatory drugs used in the on-going clinical trials for treating COVID-19 patients [52],[53].

#### 3.2.6. Adjunctive therapy

Anticoagulants such as low molecular weight heparin (LMWH) are used to treat disseminated intravascular coagulation (DIC) or thromboembolism in critically ill COVID-19 patients [54]. Laboratory features such as prolonged prothrombin time, activated partial thromboplastin time, elevated D-dimer, and fibrin degradation products are indicators for thrombotic complications requiring anticoagulant therapy [55]. LMWH acts by effectively inhibiting activated factor X along with thrombin inhibition. It also reduces IL-6 release, increases circulating lymphocytes, and improves the overhaul outcome in critically ill COVID-19 patients [56].

Vitamin C is an essential co-factor, antioxidant, and immunomodulator. In COVID-19 patients, it enhances lymphocyte proliferation, clears reactive oxygen species (ROS), and provides antiviral effects [57]. Intravenous vitamin C infusion prevents the development of cytokine storm syndrome, thereby reducing the onset of ARDS in critically ill COVID-19 patients [58]. In addition to these effects in adults, it is also known to reduce symptoms and mortality in children [59].

### 3.3. Potential COVID-19 Vaccines

The rapid development, distribution, and administering of a vaccine is the most effective way to fight this pandemic. A global attempt is being made to expedite the development of a vaccine against the SARS-CoV-2. Due to the outgrowing pandemic, more than 150 candidate SARS-CoV-2 vaccines were in development within the first five months of 2020 [60].

Due to the asymptomatic transmission of COVID-19, a vaccine is crucial in disease containment [61],[62]. Scientists started working very early on developing inactivated or attenuated viral vaccines along with subunit vaccines for prophylaxis and treatment. The first vaccine candidate launched into clinical trials is an mRNA vaccine was delivered via lipid nanoparticles [61].

The genome and structural information of SARS-CoV-2 was made available very early in the pandemic [63],[64]. Earlier development of SARS/MERS vaccine candidates has helped develop the current SARS-CoV-2 vaccine [65]. The vaccine aids in gaining knowledge on structural information, and researchers featured the full-length S protein, S1, RBD, and S2 subunits derivatives, which contained the prime target epitopes for the induction of neutralizing antibodies [66].

Contemporary vaccines include live attenuated, inactivated vaccines, and viral vectors. The use of a live virus or a whole pathogen in a weakened or killed form through chemical or physical processes has been traditionally relied on. Viral vectors like the herpes simplex virus have also been used.

#### 3.3.1. Live Attenuated Vaccines (LAVs) and Inactivated vaccines (IVs)

LAV is a live but avirulent form of virus vaccine. LAVs have a huge potential to become the first choice of vaccine candidates for the COVID-19 pandemic due to their long-lasting experience. However, they have certain disadvantages: the most prominent one is the requirement of cold chain distribution. As a result, they possess the challenge of loss of efficacy and reproductive potential. Therefore, there are new evolving technologies such as genetic code expansion and synthetic genomics. Strategic use of recombinant SARS-CoV-2 viruses from fragments of viral DNAs could be applied towards the generation of SARS-CoV-2 LAVs [67],[68]. IVs are developed by thermal or chemical inactivation of pathogens. They are safer than LAVs as they are incapable of replication. Their disadvantages include its low immunogenicity, need for multiple-dose regimens for long-lasting immunity, and also the requirement of cold chain distribution like LAVs. Sinovac was recently approved to perform its clinical trial for COVID-19 IVs.

#### 3.3.2. Viral vectors

There are a couple of viral vector vaccine candidate evolving. CanSino Biological has utilized an adenovirus type-5 vector (Ad5-nCoV) as of March 16, 2020, and the University of Oxford has used a chimpanzee adenovirus vaccine vector (ChAdOx1) as of March 31, 2020. Some of the advantages of adenoviral vectors are adjuvant qualities, scalability, and their broad tissue tropism. Disadvantages include pre-existing immunity in humans, which can decrease the efficacy of the vector. Therefore ChAdOx1, which has low human seroprevalence, has been used as an alternative vaccine platform [69],[70].

#### 3.3.3. Next-generation vaccines

Viruses can be considered as a nanomaterial. LAVs, IVs, and viral vectors are all examples of nanotechnologies. Nanotechnology approaches in vaccine development and immune-engineering are extremely powerful. The reason being both nanoparticles and viruses operate at the same length scale. Nanoparticles, including both natural and synthetic, are similar to the structural features of viruses. The invention of next-generation designer vaccine technologies is based on chemical biology, biotechnology, and nanochemistry [71],[72].

#### 3.3.4. Nucleic acid-based

Under this category, both DNA and mRNA vaccines are included. These vaccines are very reliable when it comes to safety, stability, speed, and scalability. They have antibodies, CD 4 + T cell response, and CD 8 + cytotoxic T cell response to eradicate the virus [73]. However, they have an increased risk of failure in clinical development than other novel technologies. Unfortunately, there is no licensed DNA or RNA vaccine. Inovio Pharmaceuticals and Entos Pharmaceuticals, Inc., based in Alberta, Canada, started their Phase I clinical trial in April 2020. In the U.S., phase I clinical trials for mRNA based technology was started by Moderna on March 16, 2020. A recent announcement was made by Biotech-Pfizer on the Phase I/II clinical trial regulatory approval in Germany to test for four lead mRNA vaccine candidates [74]. The mRNA vaccines have no risk for insertional mutagenesis. The trial to prolong the short half-life of the RNA and step up S protein expression levels is conducted by researchers at the Imperial College of London and Arcturus Therapeutics. There are several synthetic carriers in the form of cationic liposomes and polymeric nanoparticles. Moderna's mRNA vaccine is based on a lipid nanoparticle platform. Other nanotechnology platforms for improving the delivery of mRNA-based vaccines include cationic nanoemulsions, liposomes, dendrimers, or polysaccharide particles [75].

#### 3.3.5. Subunit vaccine

This category comprises protein or glycoprotein components of a pathogen capable of inducing a protective immune response and may be produced by conventional biochemical or recombinant DNA technologies. Novovax initiated a Phase I/II trial on May 25, 2020. Molecular Clamp Technology and the Trimer-tag technology are being used to develop subunit vaccines by Clover Biopharmaceuticals [76] and the University of Queensland. Medicago and iBio have commenced their clinical trials in July 2020 and are using Nicotiana benthamiana to produce VLPs using the S protein. AdaptVac/ExpreS2ion is using an insect cell expression system to make VLPs from the S2 protein. Sanofi Pasteur/GSK, Vaxine, Johnson & Johnson, and the University of Pittsburgh have announced that they expect to begin Phase I clinical trials shortly [77].

#### 3.3.6. Peptide-based vaccines

This is an immunotherapy-based technique wherein a peptide is applied using an immuno-adjuvant (nanoparticle or biopolymers) to stimulate T-cell and B-cell immunity. However, it has been observed in earlier studies of SARS and MERS vaccine that there might be a risk of antibody-dependent enhancement (ADE) of infection. It has also been suggested that high IgG titers correlate with worse outcomes. Peptide-based vaccines represent the simplest form of efficiently designed vaccines, readily validated and rapidly manufactured. Several plant-based nanotechnologies for cancer vaccines and immunotherapy have been developed effectively. This technology is being used towards the development of COVID-19 vaccines just to ensure that the next wave of SARS-CoV-2 infections and other emerging viruses are met with a more efficient and rapid response [78].

Vaccine development includes 3 phase clinical trials, namely, phase I (safety test), phase II (efficacy and adverse effect), and phase III (large scale clinical trial). The early development phase includes identifying antigens based viral proteins by genetic sequencing. An animal trial follows this, and if successful, the human trials are initiated to procure a successful candidate vaccine [79],[80].

A targeted approach in generating both cell-mediated immune response and antibody production by introducing viral proteins is considered the most effective vaccination strategy against COVID-19. The S and N proteins of SARS-CoV-2 are short-lived and are associated with major histocompatibility complex (MHC) in the human population [81]. These, along with many B-cell epitopes identified on S protein domains, indicate possible access to antibodies following virus-host interaction. The other important factor to be considered is the presence of antigenic drift involved in genetic sequencing during the vaccine development phase. This requires large scale genomic sequencing across various regions worldwide to identify its mutation and genetic variability [82].

An efficient vaccine against SARS-CoV-2 development is governed by various factors such as the disease attack rate and the inclusion of vulnerable populations in the study. The vulnerable population in COVID-19 are the elderly and adults with comorbidity. However, due to patient safety regulations, this group is usually avoided in the on-going trials posing sample size challenges and the developed vaccine's quality [83].

Challenges in the development of the CoV vaccine are that the animal models have shown an immunogenic response to SARS-CoV-2. Still, they have failed to prevent disease acquisition, to induce long-lived immunity, and have caused various levels of safety concerns. Vaccine-associated disease enhancement should also be monitored [84].

### 3.4. Current status of COVID-19 vaccines

A large trial that included 43,548 participants from 152 sites globally, most of which were in the U.S., has been reported recently. BNT162b2, a nucleoside-modified RNA which is lipid nanoparticle-formulated and encoding the SARS-CoV-2 full-length spike, modified by two proline mutations when administered as a two-dose regimen (30 µg per dose, 21 days apart) in adults 16 years of age or older who were healthy or had stable chronic medical conditions were found to be safe, and 95% effective against COVID-19 [85]. No death was reported attributable to the vaccine. The majority of the adverse events were reported as reactogenicity events and transient. U.S. phase 1 trial of two vaccine candidates, BNT162b1 and BNT162b2 in younger and older adults, has been completed and has added to earlier interim safety and immunogenicity data in Germany and the US [86]. Another messenger RNA vaccine, mRNA-1273, also has completed phase I trial [87]. They are getting advanced to a pivotal phase 2–3 safety and efficacy evaluation.

### 3.5. BCG vaccination and COVID-19

Trained immunity, which is a process of epigenetic, transcriptional, and functional reprogramming of immune cells (innate such as myeloid or N.K. cells), leads to an increase in the cytokine production capacity and their anti-microbial function. Since the COVID-19 specific vaccines take time to be available, Bacille Calmette-Guérin (BCG), oral polio vaccine (OPV), or the measles vaccine were suggested to bridge the gap in the meantime. The BCG vaccination was particularly tried in the ACTIVATE trial wherein 198 elderly adults were randomized to receive it or the placebo. The infection rate was 17% lower in the BCG arm after a follow up for 12 months [88]. Another large trial recruiting 800 participants has started in Brazil, with recruitment being completed by March 2021. The follow-up is for six months; the results will likely be available by the end of this year [89]. A recent study among a cohort of health care workers concluded that a history of BCG vaccination has been associated with a decrease in anti-SARS-CoV-2 IgG seroprevalence and a decrease in the number of participants self-reported with COVID-19-related clinical symptoms in this cohort [90].

## 4. Conclusions

COVID-19 has caused a serious global health concern due to its rapid spread, high morbidity, and economic challenge in the health sector across numerous countries. Early diagnosis, effective treatment, and preventive measures form the cornerstones in disease containment. A rapid and specific diagnostic approach is essential in identifying COVID-19 positive cases. This would allow prompt isolation and early treatment initiation to such patients in designated centers. Treatment strategies vary depending on the severity of illness and symptom manifestation. There is also an urgent need for prospective clinical trials of BCG vaccination to confirm whether BCG vaccination can have a protective effect against SARS-CoV-2 infection. Due to numerous challenges encountered across many countries in controlling COVID-19 through existing preventive measures, an effective and economical vaccine remains the only retort to combat COVID-19 successfully.
